# Similar effects as shade tolerance induced by dust accumulation and size penetration of particulates on cotton leaves

**DOI:** 10.1186/s12870-021-02926-6

**Published:** 2021-03-23

**Authors:** Li Li, Guijin Mu

**Affiliations:** 1 State Key Laboratory of Desert and Oasis Ecology, Xinjiang Institute of Ecology and Geography, Chinese Academy of Sciences, Urumqi, 830011 China; 2 Xinjiang Desert Plant Roots Ecology and Vegetation Restoration Laboratory, Xinjiang Institute of Ecology and Geography, Chinese Academy of Sciences, Urumqi, 830011 China; 3 Cele National Station of Observation and Research for Desert-Grassland Ecosystems, Cele, 848300 China

**Keywords:** Foliar dust retention, Photoinhibition, Nano-fluorescent microspheres, Low light acclimation, Stomatal penetration

## Abstract

**Background:**

Dust accumulation covers the leaf’s surface and influences foliar physiological activity. Two independent experiments were carried out to instigate the foliar responses to dust accumulation and the penetration limitation of small dust particles (< 1 μm) on the foliar surface, respectively. In experiment I, three dust accumulation intensities were achieved by a dust spraying treatment. Photosynthesis CO_2_ exchange and fast chlorophyll fluorescence transient were measured, as well as chlorophyll contents and leaf thickness. In experiment II, the penetration limits of small particulates on the leaf surface were examined by feeding nano-fluorescent microspheres.

**Results:**

Dust accumulation alleviated the photoinhibition of Photosystem II and decreased photosynthesis, as represented by net photosynthetic rates (*P*_N_) and stomatal conductance to water vapor (*g*_s_). Photosynthetic response curves between net photosynthetic rate (*P*_N_) and photosynthetically active radiation (PAR) showed that heavy dust accumulation (34.98 ± 2.6 mg cm^− 2^) increased the light compensation point (LCP) and light saturation point (LSP) and decreased photosynthesis rates under saturating light (*P*_Nmax_). Leaves became thin due to the lack of a palisade layer while chlorophyll content increased under dust accumulation. Confocal laser scanning microscopy (CLSM) images showed that the larger particles (1 μm) distributed in the regions below the stomata and the smaller ones (0.1 μm) were detected in the wider areas below stomata.

**Conclusions:**

These results suggested that dust accumulation induced similar effects as shade tolerance in cotton leaves but did not trigger more photochemical acclimation to low light. Dust particles (< 1 μm) penetrated leaf surface through stomata.

**Supplementary Information:**

The online version contains supplementary material available at 10.1186/s12870-021-02926-6.

## Background

Dust suspension and transport in air is a common phenomenon. Dust particles can deposit on leaves when wind velocity is slow [1]. Plants can intercept and gather dust particulates on leaf surfaces [2]. Dust quantity and distribution on leaf surfaces are affected by foliar characteristics [3]. In turn, the foliar retention of particulate matter changes traits of the leaf surface interface, causing variation in the biochemical and physiological function of leaves. Previous studies reported that dust accumulation changed surface anatomy and morphological structure [4], decreased photosynthetic assimilation rates [5], stomatal conductance [6], activity of Photosystem II (PSII) [7], and produced membrane injury [8]. Even heavy dust cover led to individual death due to extreme suppression of photosynthesis [6, 9]. Moreover, dust accumulation on the leaf surface indirectly causes secondary stresses, such as drought or pathogen attack [10, 11]. The effects of dust accumulation on leaf surfaces include a series of direct and indirect consequences. Toxic effects of the compounds carried by atmospheric particulate matter on leaf physiology and growth have been wildly reported [12–14]. However, those basic mechanisms of dust effect remain unclear.

Light is an indispensable and crucial environmental factor and provides an energy resource for photosynthesis. Light quantity and quality can deeply impact plant performance [15]. The variation in light environments can induce a series of light adaption changes to physiological and morphological characteristics. Low-light irradiation drives plants’ two adaption strategies: shade tolerance and shade avoidance [16]. Shade tolerance occurs when shade is an unavoidable pressure, for example, underneath a leaf canopy. Under unavoidable low light, plants tend to strengthen those features optimizing light capture [15], such as a bigger specific leaf area (SLA) and a higher chlorophyll concentration [17, 18]. Lower light levels still cause growth to decrease, such as by the photosynthetic rate and stomatal conductance [19]. Dust accumulation had been thought to reduce the light intensity that reaches photosynthetic tissues [6, 20, 21]. The short-term effects of dust accumulation on cotton leaves in the Tarim Basin of China included alleviation of photoinhibition induced by high radiation in PSII [22]. At the foliar level, acclimation may be displayed through morpho-anatomical and/or physiological adjustments in response to changes in the light environment. Therefore, we speculate that leaf traits adapt as they would for shade tolerance when dust accumulation occurs long term.

Atmospheric particulate matter can accumulate near the stoma and block the cavity, causing the closure of stoma [21]. Shrinking of the stomatal opening is responsible for a decrease in stomatal conductance [23]. In addition, nanoparticles have been reported to penetrate and be transported within living plants [24]. However, the effects of particulate size are often ignored. The length of stomata is in the range of several to a dozen micrometers. In principle, smaller particulates (e.g., < 1 μm) may pass through stomatal apertures and enter the cavity. In order to confirm this speculation, we chose nano-fluorescence particles (1 and 0.1 μm) to examine the effects of small particulates on stomata.

In the present study, our aims were to investigate the effects of dust accumulation intensity (light, medium and heavy) on the anatomy and physiology of cotton leaves at the foliar level to confirm the hypothesis that dust accumulation causes shade tolerance effects. Additionally, we examined the penetration limits of small particulates on stomata.

## Results

### OJIP fluorescence transients

**Fig. 1:**
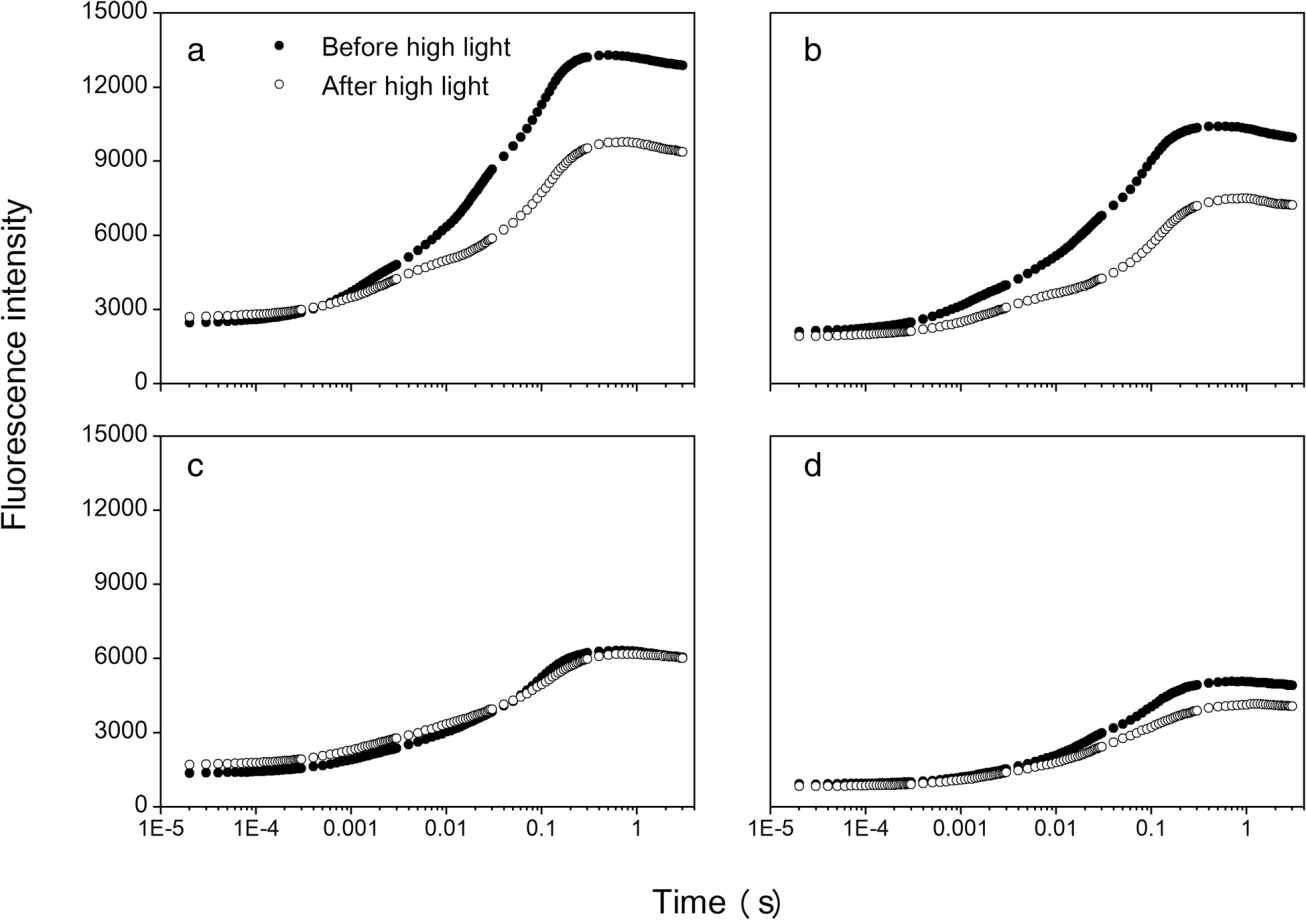
Transient rise curves of cotton leaves in the control **a**, and after light **b**, medium **c**, and heavy **d** dust accumulation. X-axis are in the log scale. Each value is a mean of four or five replicates

**Fig. 2:**
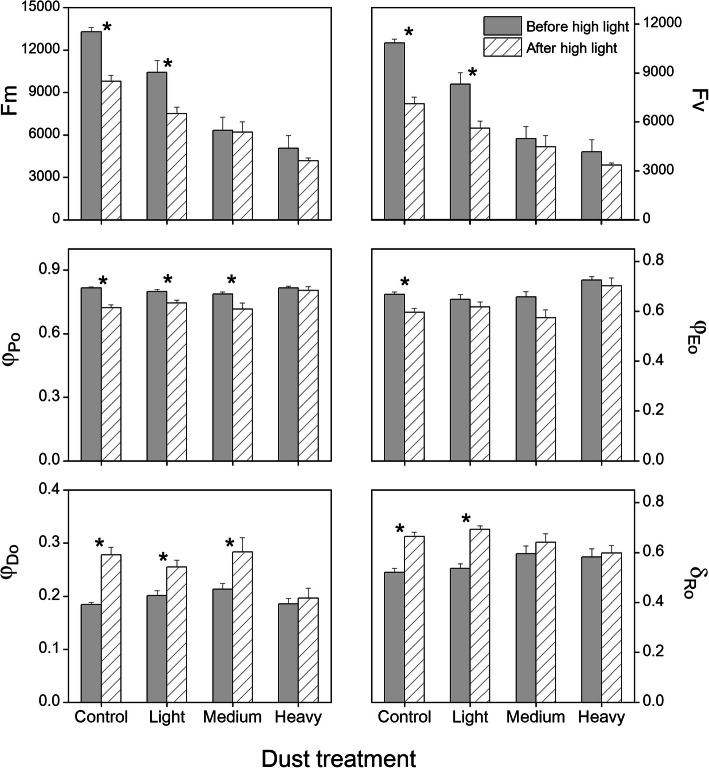
The selected fluorescence parameters of cotton leaves in the control, and after dust accumulation (light, medium, and heavy). The values shown are the mean of five replicates. Bars represent the standard error. Asterisks (*) indicate significant differences between before and after high-light treatments at 0.05% level

Regardless of light intensity, fast fluorescence transient curves showed a significant decrease as the dust accumulation intensity increased (Fig. 1). In general, F_m_ decreased more than F_o_. Considering light intensity, there was a higher intensity of chlorophyll fluorescence after high light (> 1200 μmol m^− 2^ s^− 1^ PPFD for 30 min) than before high light; the differences decreased as the degree of dust accumulation increased, and there was no difference between MD and HD treatments. The two curves of high-light treatments almost coincided under MD and HD treatments (Fig. 1). In detail, differences between before and after high light under LD and the control were attributed to the reduction in F_m_, while F_o_ remained unchanged. JIP-test analysis showed more information (Fig. 2). High light decreased F_v_, φ_Po_, φ_Eo_, and δ_Ro_, and increased φ_Do_ under the control and LD treatments but no difference was observed under MD and HD dust accumulation.

### Photosynthesis CO_2_ exchange

**Fig. 3:**
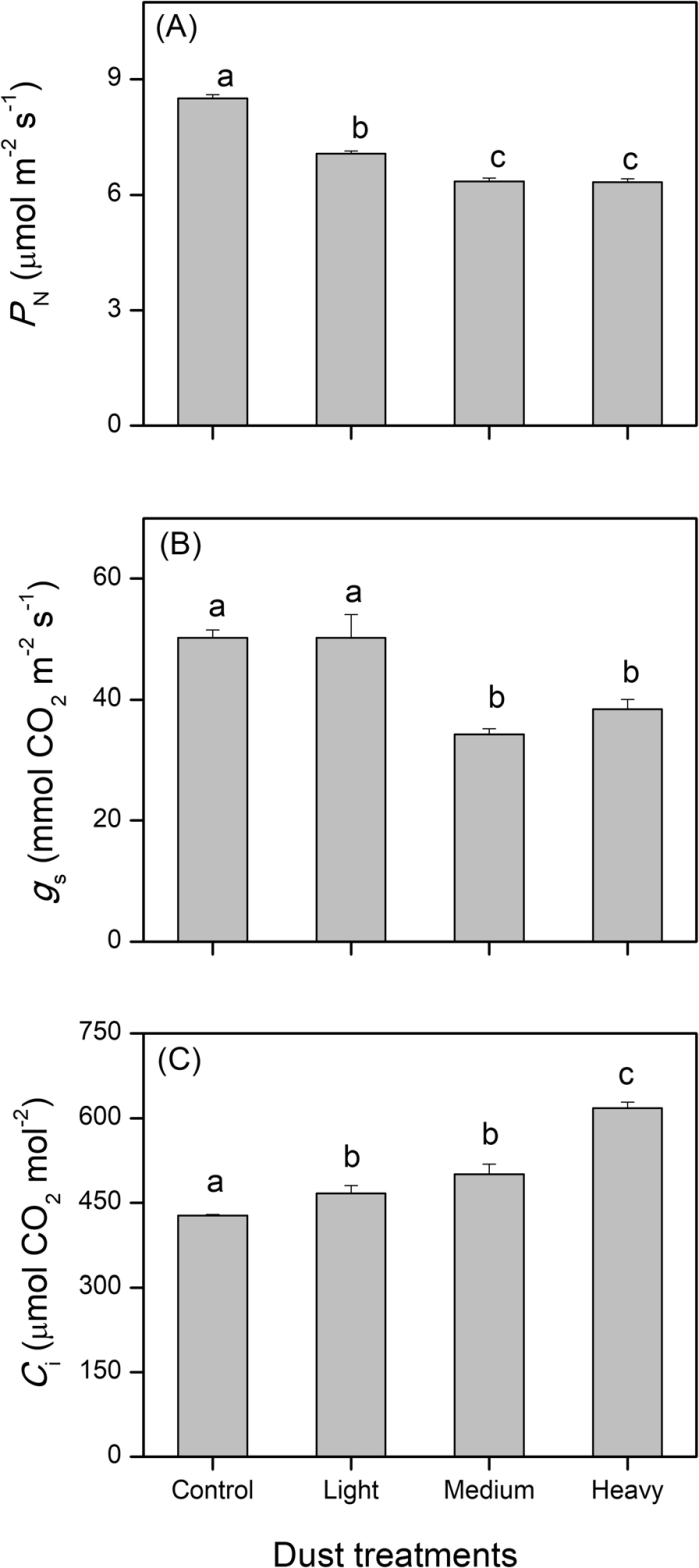
Gas exchange parameters of cotton leaves in the control, and after dust accumulation (light, medium, and heavy). The values shown are the mean of six replicates. Bars represent the standard error. Different letters indicate significant differences among the dust treatments at 0.05% level. *P*_N_: net photosynthetic rates; *g*_s_: stomatal conductance to water vapor; *C*_i_: internal CO_2_ concentration

Dust accumulation decreased *P*_N_ and *g*_s_, but increased *C*_i_ (Fig. 3). *P*_N_ significantly decreased in all dust treatments. *P*_N_ under MD and HD treatments dropped to 74.75% and was only 74.51% compared with the control. LD treatment had no effect on *g*_s_ compared with the control, but its value under MD and HD was reduced 31.65 and 23.35%, respectively, compared with that of the control, with no significant effect between MD and HD treatments. *C*_i_ increased as the intensity of dust accumulation increased. Its values under HD treatments rose to almost half of the control (44.5%) (Fig. 3).

### Photosynthetic responses to light

**Fig. 4:**
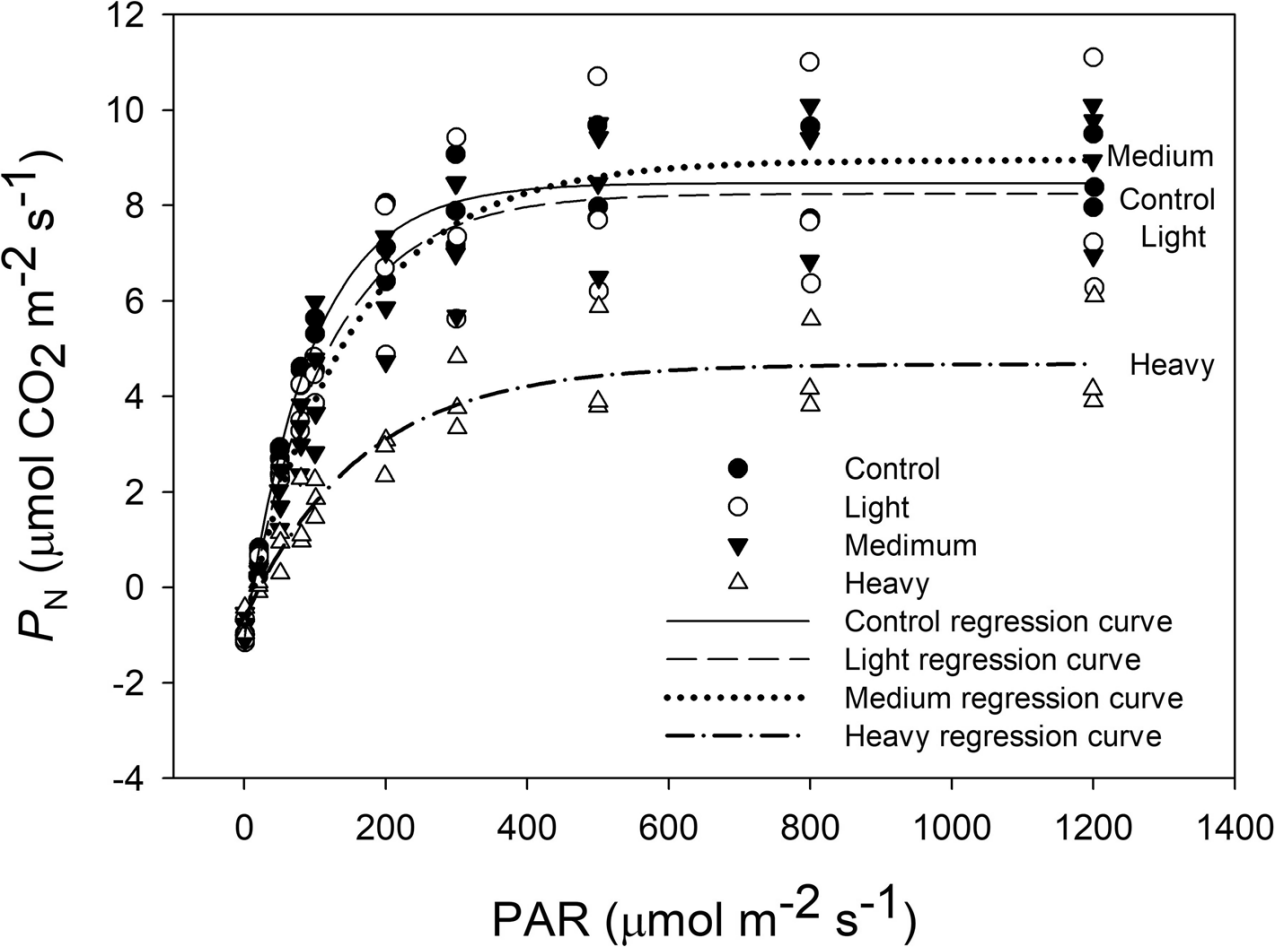
Effect of PAR on net photosynthesis rate (*P*_N_) of cotton leaves after one-month of dust accumulation (*n* = 4 plants per treatment)

Light response curves of photosynthesis (*P*_N_/PAR) in cotton leaves covered by dust for 1 month are shown in Fig. 4, and the related fitting parameters are summarized in Table 1. The three fitted curves of the control, and LD and MD treatments had similar trends, but the curve of the HD treatment had a significant decrease (Fig. 4). In the estimated photosynthesis parameters, in LD and MD treatment and the control, there were no significant differences in LSP, LCP, and *P*_Nmax,_ except for LSP under MD treatment, which showed a significant increase. Conversely, all of the parameters under HD treatment were significantly different compared with the other treatments. Leaves under HD treatment had significantly higher LSP and LCP, and lower *P*_Nmax_ compared with those of the control (Table 1).

**Table 1:** Parameters in light response curves when cotton leaves were covered in dust (light, medium, and heavy) or the control

Treatments	Control	Light	Medium	Heavy
LSP (μmol m^− 2^ s^− 1^)	228.06 ± 14^a^	271.09 ± 36^a^	366.18 ± 23^b^	396.07 ± 33^b^
LCP (μmol m^− 2^ s^− 1^)	11.70 ± 0.41^a^	13.10 ± 1.79^a^	14.38 ± 1.04^a^	22.29 ± 1.44^b^
*P*_Nmax_ (μmol CO_2_ m^− 2^ s^− 1^)	8.47 ± 0.59^a^	8.27 ± 1.44^a^	8.96 ± 0.72^a^	4.68 ± 0.66^b^

*P*_Nmax_ light-saturated net photosynthetic rate, *LSP* light saturation point, *LCP* light compensation point. Values are shown as the mean ± standard error (*n* = 4 or 5). The different letters in superscripts indicate significant differences among treatments at 0.05% level

### Leaf thickness and specific leaf area (SLA)

**Fig. 5:**
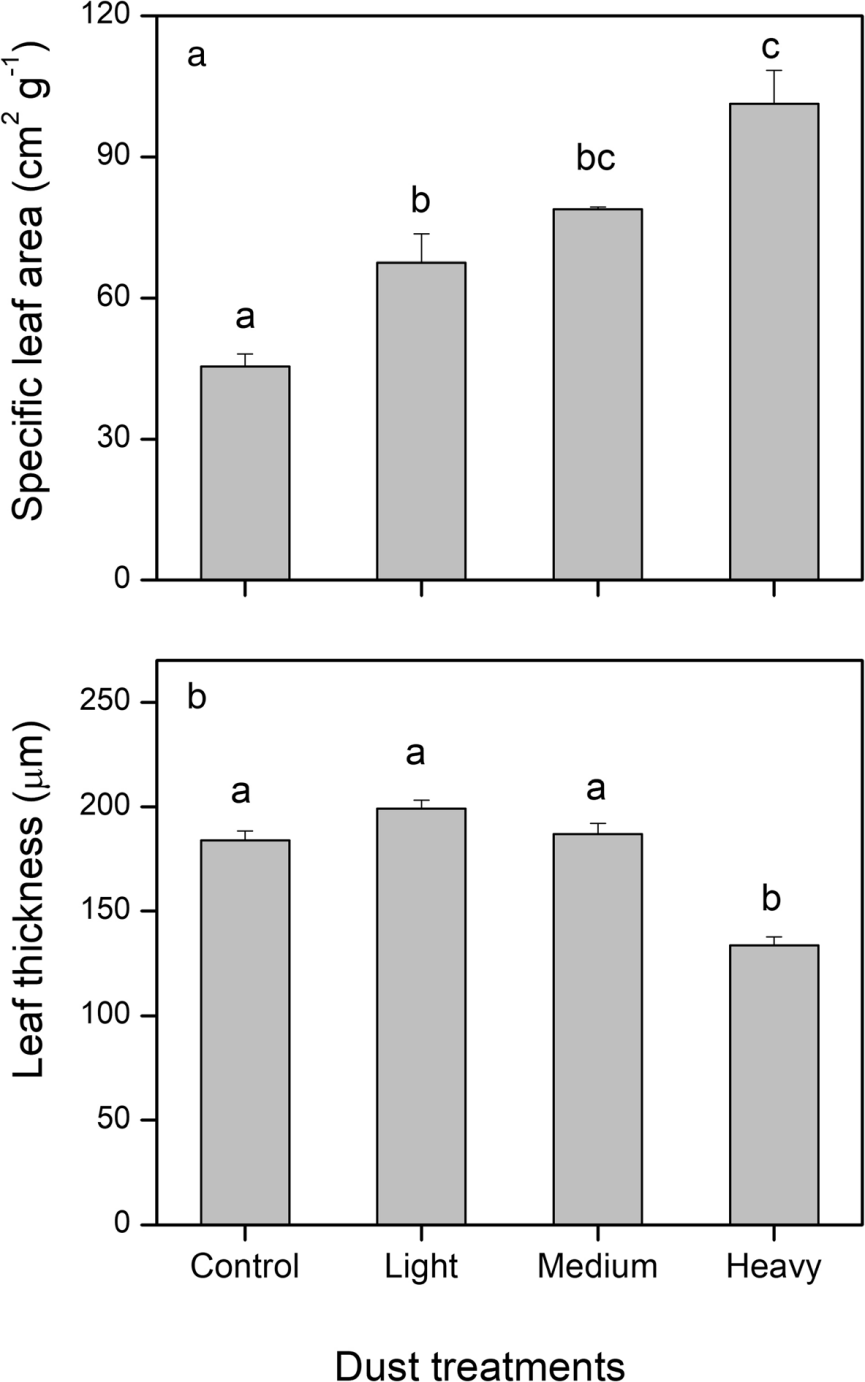
Specific leaf area (cm^2^ g^− 1^) **a** and leaf thickness (μm) **b** of cotton leaves in the control, and after dust accumulation (light, medium, and heavy). The values shown are the mean of 4 replicates. Bars represent the standard error. Different letters indicate significant differences among the treatments at a 0.05% level

**Fig. 6:**
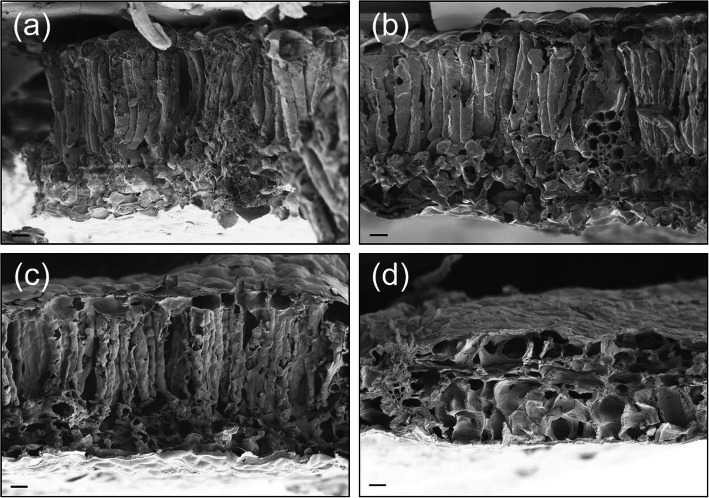
SEM images of leaf cross-sections under different dust treatments: the control **a**, light **b**, medium **c**, and heavy **d** accumulation. Horizontal bars represent 20 μm

Specific leaf area (SLA) increased as the intensity of dust accumulation increased, but leaf thickness showed the opposite trend and significantly decreased under HD treatments (Fig. 5). SLA after dust accumulation was significantly higher than the control and were 48.6% for LD, 73.8% for MD, and 123.2% for HD (Fig. 5a). Leaf thickness had no significant effect on LD and MD treatments and the control. Under HD treatments, leaf thickness was 72.6% of the control and showed a significant decrease in comparison to the control (Fig. 5b). The results were confirmed by the SEM images of leaf cross sections (Fig. 6). More information on the anatomy was exhibited through SEM. The leaf cross section displayed the compact palisade mesophyll tissues for the control and LD and MD treatments, but full of big spongy mesophyll cells instead of palisade tissue was observed for those leaves under HD treatment (Fig. 6).

### Leaf chlorophyll contents

**Fig. 7:**
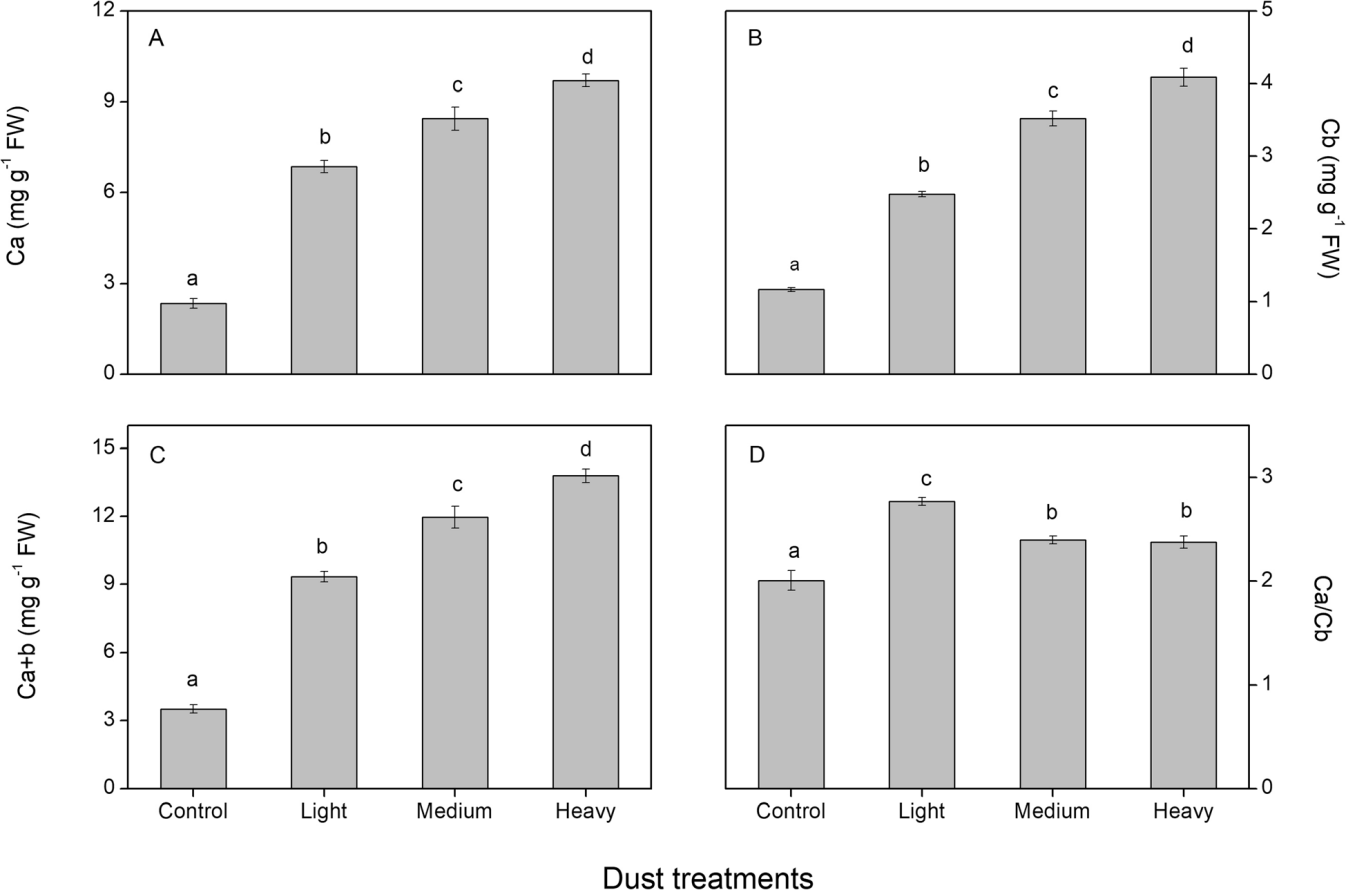
Effect of dust treatments on chlorophyll content (mg g^− 1^ fresh weight) in cotton leaves. Ca , Cb , Ca + b and Ca/Cb represented the contents of chlorophyll a, Chlorophyll b, total chlorophyll content, and the ratio of chlorophyll a and chlorophyll b, respectively. The values shown are the mean of four replicates. Bars represent the standard error. Different letters indicate significant differences among the treatments at a 0.05% level

Chlorophyll content gradually increased as the intensity of dust accumulation increased (Fig. 7). The three contents, chlorophyll a, chlorophyll b, and total chlorophyll content, had similar variation. The lowest content was obtained in the control and the highest in the HD treatment. Compared with the control, values under HD treatment were 3.1-fold higher for Chlorophyll a, 2.5-fold for Chlorophyll b*,* and 2.9-fold for total chlorophyll content. The Chlorophyll a/b ratio for LD treatments was highest. MD and HD treatments were lower than LD and higher than the control but were not significantly different from each other.

### Stomatal occlusion and penetration

**Fig. 8:**
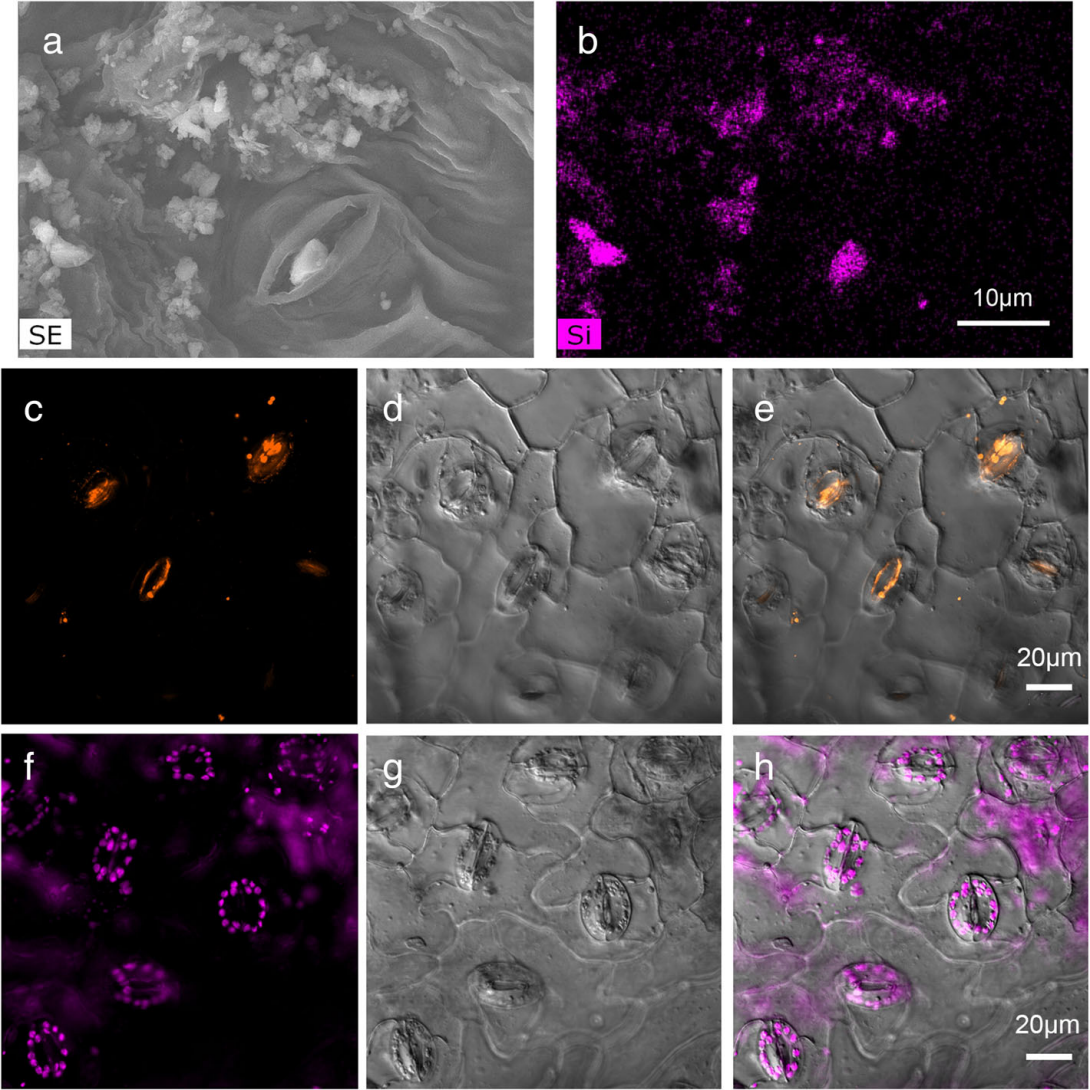
An occlusive stoma by dust particles on the adaxial surface in cotton growing in the field under SEM **a** and EDS **b** images. The energy-dispersive spectrometer (EDS) showed the Si content. The scale is 10 μm. CLSM images of the foliar surface after 20 days by feeding 1 μm **c**, **d**, **e** and 0.1 μm **f**, **g**, **h** carboxylate-modified fluorospheres in 1-week-old cotton seedlings. C and F show fluorescence, D and G show SEM, and E and H are overlaps of the corresponding fluorescence and SEM images. The scale shown is 20 μm

A SEM image of stomatal occlusion by dust particulate matter is showed in Fig. 8a. The corresponding EDS image confirmed the dust particle component of Si (Fig. 8b). The dust particle, with abundant Si content, plugged the stomata. The cotton leaf was collected from the field in Cele station in the southern fringe of Tarim Basin in China.

A 20-day-experiment of cuticle penetration was carried out by application of suspensions of fluorescent particles. Two particle sizes (0.1 and 1 μm diameter) were used to imitate the foliar spraying of dust particles. The CLSM images (Fig. 8c-h) shows that more 0.1-μm particles penetrated the cuticle and stoma and entered into the internal system than 1-μm particles. In addition, 1-μm particulate matter mainly distributed in the stomatal cavity and 0.1-μm particle distributed more widely under the cuticle.

## Discussion

In this study, PSII responses to different dust accumulation intensities were instigated by the fast chlorophyll transient measure under before and after high-light conditions. Chlorophyll fluorescence was a fast and nonintrusive way to detect foliar responses to environmental conditions [25]. As a whole, dust accumulation depressed the intensity of chlorophyll fluorescence under before and after high light. The low fluorescence values induced by dust accumulation may result from decreasing the light intensity that reached photosynthetic tissues [21]. When compared between light treatments, high light reduced F_m_, F_v_, φ_Po_, and φ_Eo_ while it increased δ_Ro_ and φ_Do_ in the control and LD treatments, which indicated the presence of high-light inhibition. Excess light energy inhibited the quantum yields of photo induced electron transport in the PSII reaction center to Q_A_ (φ_Po_) and from Q_A_^−^ to plastoquinone (φ_Eo_), and more energy was dissipated by heat [25] or transferred into PSI end electron acceptors [26]. Thus, overexcitation energy pressure in PSII can be released objectively [27]. Under HD treatment, no difference in fluorescence parameters suggested the absence of inhibition under before and after high light, confirming the decrease in light intensity on the foliar surface. Similar results have been obtained with dust accumulation on cotton leaves in the Tarim Basin for short-term experiments [22]. Generally, heavy dust accumulation reduced the photochemical efficiency of cotton leaf, which was consistent with the effect of heavy shade on four conifer species [17].

Light performs an important role in photosynthetic efficiency and biomass accumulation. In general, lower light decreased the photosynthesis rate [28, 29] and stomatal conductance [30] when light intensity was lower than the light saturated point. This is supported by our present findings that dust accumulation decreased *P*_N_ and *g*_s_, suggesting similar responses between shade and dust covered leaves. In addition, dust accumulation increased *C*_i_ of cotton leaves. Similar results were obtained by Lavinsky et al. [31] in photosynthetic acclimation in shade-developed leaves of *Euterpe edulis* (Arecaceae). The accumulation of *C*_i_ may be due to a decrease in *g*_s_. When a decrease in *g*_s_ and increase in *C*_i_ occurred simultaneously, the decrease in P_*N*_ can be attributed to nonstomatal limitations [32]. So, the decreased photosystem activity upstream down-regulated photosynthesis assimilation and gas exchange downstream.

Under light-limiting conditions, LCP is a key factor for plant survival and growth. Kitao et al. [33] found that shade induced a lower LCP to improve the light absorption and availability under light-limiting conditions. Shade decreased LSP and LCP [34, 35]. However, we obtained inconsistent results that dust accumulation increased both LSP and LCP. Still, heavy dust accumulation decreased photosynthesis rates under saturating light (*P*_Nmax_). *P*_Nmax_ represents the resource capture ability of leaves and relies, not only on photosynthetic biochemistry, but also on the mesophyll structure of leaves [36]. This suggests that dust accumulation reduced the sensitivity to light in cotton leaves but did not change the photosynthetic mechanism to light intensity. Therefore, cotton leaves respond to heavy dust accumulation for 1 month through morphological and physiological adaption, rather than biochemical acclimation of the photosynthetic apparatus.

Plants have relatively high phenotypic plasticity in response to light intensity [37, 38]. Low-light availability induced thinner leaf thickness and lower leaf area per unit of dry mass, which was expressed as specific leaf area (SLA) [39]. The increase in SLA might often be related to the decrease in leaf thickness. Thinner leaf thickness was attributed to the lack of a palisade layer or shorter palisade cells [40]. These conclusions were supported by our results. In the present study, an increased dust accumulation led to an increased SLA. Heavy dust accumulation remarkably thinned the leaf thickness. Observations in SEM images showed that the lack of a palisade layer was responsible for the thinner leaves under heavy dust accumulation. Leaf thickness and structures affect CO_2_ absorption. Thinner leaves favor CO_2_ diffusion, due to shorter length between the stomata to mesophyll cells and chloroplasts [41]. Foliar variation induced by dust accumulation would be advantageous for light absorbance, which is consistent with that under low-light environments [42].

Chlorophyll is an important component in photosynthetic systems and plays a key role in determining foliar absorbance. Under low-light environments, plants increase the contents of chlorophyll. The increase in the number of chloroplasts can enhance the photosynthetic capacity per unit leaf area [43]. Present studies showed that dust accumulation increased the total chlorophyll, chlorophyll a and b contents, which were highest in HD, lowest in the control, and intermediate in LD and MD. A higher chlorophyll content represents higher leaf absorption, which is often related to shade tolerance [33, 42]. Chlorophyll b content was considered an indicator of acclimation to shade due to its role in the light-harvesting complex [44]. In this study, alterations in chlorophyll content suggested similar responses to dust accumulation as to shade acclimation. Chlorophyll a/b ratios were sensitive to both light quality and quantity [45] and exhibited a decrease in shade leaves [18], which was confirmed to facilitate light interception [19, 36]. However, our studies had the opposite effect; dust accumulation increased the Chlorophyll a/b ratio. A similar result was reported by Jiang et al. [46] on physiological acclimation of seashore *paspalum* and bermudagrass to low light. Mendes et al. [30] observed that the Chlorophyll a/b ratio was not affected by light intensity. Therefore, species-specific traits to shade tolerance may be responsible for the differences in Chlorophyll a/b ratio [45].

Leaf surfaces can gather dust particles. The dust distribution pattern had a close relationship with the foliar surface structure [3]. Atmospheric particulate matter was reported to accumulate near stomata and occlude the stomatal aperture [7]. Similarly, we found stomata plugged by dust particles after observing several thousand stomata.

The epidermis is composed of waxes and compact matter that protect plants from water loss and external damage [47]. Some material can enter and be taken up into the plant by the cuticular and stomatal pathways [48]. Nanoparticles were reported to be capable of penetrating living plant tissues and moving to different regions of the plant [24]. Supporting these results, our experiment with carboxylate-modified fluorescent microspheres on the cotton leaf surface showed that two kinds of microspheres (1 and 0.1 μm) can penetrate the leaf surface and enter mesophyll cells. The larger particles (1 μm) distributed in the regions below the stomata and the smaller ones were detected in the wider areas below stomata. This suggested that the 1 μm microspheres penetrated through the stomata but 0.1 μm microspheres may penetrate two channels, through the stomatal and cuticular pathway. Considering the stomatal size, we thought that it was possible for both microspheres (0.1 and 1 μm) to penetrate through the stomata. Our conclusions support the stomatal uptake of water-suspended nanoparticles < 1.1 μm observed in Eichert et al. [48].

Dust particles dry-deposited on the leaf surface have different physical characteristics than carboxylate-modified fluorescent microspheres that are suspended in a diffusion medium. In the natural environment, however, the leaf surface often becomes water-saturated when influenced by rain, dew, and phyllosphere microorganisms. Thus, small dust particles have a high potential to penetrate leaf cells through water suspensions of dust particles [49]. Foliar uptake of atmospheric dust particles (nanoparticles), as inert dust or other materials, should be observed in pollutant studies.

Rapid pace of industrialization and urbanization in global scale increases atmospheric particulate matter, a pervasive pollutant [10]. Deposition of particles on leaf surfaces is likely to change foliar traits and ecophysiological functions. In those areas with high radiation, foliar dust accumulation might intercept light and alleviate photohibition, which is advantageous for growth to some extent. However, thinner leaves and lack of palisade mesophyll may also lead to declines in support and defense capabilities, increasing the environmental risks of mechanical and biological attacks such as rain blowing, insect feeding and microbe infecting. In addition, atmospheric particles often contain some toxic material such as salt crystals, heavy metals, and oxidizing materials [21]. These materials attached to dust particles (< 1 μm) may enter plant cells simultaneously, resulting in a series of secondary effects on plant metabolism.

## Conclusions

Foliar dust weakens the light intensity that arrives at the leaf surface, resulting in exposure to a shade environment. In this study, dust accumulation alleviated photoinhibition under high-light conditions. Photosynthesis, represented by *P*_N_ and *g*_s_, was decreased generally. Light response curves showed a reduced LCP and increased *P*_Nmax_. Leaves became thin due to the lack of a palisade layer while chlorophyll content increased. The larger particles (1 μm) distributed in the regions below the stomata and the smaller ones (0.1 μm) were detected in the wider areas below stomata. These results suggest that dust accumulation induced similar traits to shade tolerance in cotton but did not trigger photochemical acclimation to low light. Dust particles (< 1 μm) can penetrate the leaf surface and enter mesophyll cells through stomata.

## Methods

### Experiment 1: effect of dust quantity on leaf physiology and characteristics

#### Plant material and culturing

Sterilized cotton (*Gossypium hirsutum* L.) seeds (Xinjiang Tarim River Seeds Co., Ltd., Xinjiang, China) were sown in 18 cm pots (ϕ 16 cm) in culture medium with soil and pearlite (2:1). Plants were irrigated every day by tap water and grown in a greenhouse with a day/night temperature of 25/20 °C, a 14-h photoperiod, and a photosynthetic photon flux density of 500 μmol m^− 2^ s^− 1^.

#### Dust preparation and application

A dust sample was collected from the desert in Cele National Station of Observation and Research for Desert-Grassland Ecosystems (37°00′57″N, 80°43′45″E) in China. In order to maintain a similar content to the natural retention of leaf surfaces from dust fall, dust was collected from the top 5 cm of the soil surface. The dust sample was taken to Urumqi and kept dry until use. The mean grain size was 65.3 μm with about 6% grains < 10 μm.

Dust application was carried out after cotton seedlings grew for 45 days. A small room (2 m × 3 m × 2 m) with a -blower (40 W, 12 V, wind pressure 200 Pa) was used to imitate dust deposits in the field (*see the* Additional file 1: Appendix Fig). The blower was placed outside of the room and connected by a pipe. Sand was collected in a glass that was joined to the pipe. The blower worked long enough to confirm significant differences in dust quantity between the treatments. The treatments were dusted until all of the dust was deposited in the small room. In order to determine the dust quantity of different treatments, the second leaf on the plant was sampled and cleaned in the ultrasonic cleaner. The cleaning solution was dried, and dust was weighed. Leaves were scanned to calculate the total leaf area. Each treatment included four leaves. Dust quantity was calculated as dust weight/leaf area. Thus, the dust quantity corresponding to treatments was 2.89 ± 0.4, 16.03 ± 2.5 and 34.98 ± 2.6 mg cm^− 2^ for light (LD), medium (MD), and heavy (HD) dust accumulation, respectively. The plants with no dust spraying were considered the control.

After dust treatments, pots with cotton seedlings were transported and grown in the same conditions as before application. Measurements were carried out 30-d dust application.

#### OJIP fluorescence transients

Cotton seedlings were illuminated with high light (> 1200 μmol m^− 2^ s^− 1^ PAR) for 30 min. High-light irradiation was achieved with a 400 W metal halide lamp. Chlorophyll fluorescence measurements were recorded using a Pocket *PEA* fluorimeter (*Hansatech*, Norfolk, UK). The OJIP curves were measured before and after high-light illumination. All the samples were dark-adapted for 30 min before measurements. Each treatment was replicated five times. In the fluorescence induction measurement, dark-adapted leaves showed a polyphasic fluorescence rise after being illuminated with high-intensity actinic light. The polyphasic induction curve included four steps from ‘origin’ (O) through two ‘inflections’ (J and I) and then to a ‘peak’ fluorescence level (P). The fluorescence values of these steps were obtained at 20 μs (F_o_), 2 ms (F_J_), 30 ms (F_I_), and the maximum fluorescence (F_m_) of OJIP curve. JIP-test was used to quantify the function of PSII [25, 50]. The selected parameters were as follows: F_v_, φ_Po_, φ_Eo_, φ_Do_, δ_Ro_.

#### Photosynthesis CO_2_ exchange

The net photosynthetic rates (*P*_N_), stomatal conductance to water vapor (*g*_s_), and internal CO_2_ concentration (*C*_i_) were measured using a portable photosynthesis system Li-6400 (*Li-Cor*, Lincoln, USA). The second fully expanded leaf from the plant was chosen for each measurement. Flow speeds were controlled at 300 mol s^− 1^ and the humidity, light, and temperature conditions were consistent with environmental conditions. In the greenhouse, the temperature of 25/20 °C and a photosynthetic photon flux density of 500 μmol m^− 2^ s^− 1^ were controlled. The CO_2_ concentration and relative humidity measured by a portable photosynthesis system Li-6400 were about 750 ppm and 60%, respectively.

#### Light response curves

Photosynthetic response curves (*P*_N_/PAR) between net photosynthetic rate (*P*_N_) and photosynthetically active radiation (PAR) were performed after one-month of dust accumulation (*n* = 3 plants per treatment). Measurements were conducted on a single leaf, typically the second leaf from the apex. The leaf was placed in the *LI-6400* chamber and exposed to a CO_2_ concentration of 750 mol (CO_2_) mol^− 1^, flow speed of 300 mol s^− 1^, chamber temperature of 20 °C, and relative humidity of 60%. After 15 min of acclimation, PAR was controlled at 1200, 800, 500, 300, 200, 100, 80, 50, 20, and 0 μmol (photon) m^− 2^ s^− 1^. The duration of individual steps was set as a minimum wait time of 120 s and a maximum wait time of 200 s. CO_2_ assimilation was automatically recorded after each change in PAR. The light response curves were fitted according to the equation by Prado and de Moraes [51]:

*P*_N_ = *P*_Nmax_ *[1*-exp(−*b (PAR-I*_*c*_*)*)],

where *P*_Nmax_ is the light-saturated net photosynthetic rate, b is constant, *I*_c_ is the light compensation point (LCP), and the value of light saturation point (LSP) is calculated as PAR at 90% *P*_Nmax_ [52]. Data were analyzed using SigmaPlot (version 10.0).

#### Leaf SEM

The second leaf from the top was collected and put into a container of the freeze dryer (*Labconco* freezone2.5, Kansas, USA) immediately after rinsing with water and drying with filter paper. Fully freeze-dried samples were obtained after treated for 12 h. Then, the drying leaf samples were sputter-coated with gold and observed by scanning electron microscope (SUPRA 55VP, Carl Zeiss AG, Germany). Images were captured as digital images with the maximum number of pixels. Adobe Photoshop CS6 was used to process the images.

#### Leaf thickness and specific leaf area

The second leaf was sampled and cleaned in the ultrasonic cleaner. The leaf was scanned to obtain the leaf area. The leaf sample was weighted, dried, and weighed again to obtain the leaf fresh and dry weight. Each treatment included four replicates.

Specific leaf area (SLA) was calculated according to the rate of leaf area and dry weight. Leaf thickness was determined from the SEM photograph. The relative value of thickness was obtained and converted to length (μm) according to the magnification rate.

#### Leaf chlorophyll contents

The leaf samples were weighed and extracted in 80% (v/v) aqueous acetone. The supernatant was separated, and the absorbance was measured with a spectrophotometer. The chlorophyll contents were quantified according to Marr et al. [53].

### Experiment 2: surface penetration of fluorescent microspheres

#### Plant material and culturing

Cotton (*Gossypium hirsutum* L.) seeds (Xinjiang Tarim River Seeds Co., Ltd., Xinjiang, China) were planted in 8 cm pots (ϕ 5 cm) in culture medium with soil and pearlite (2:1) and grown in a controlled environment chamber with a day/night temperature of 28/20 °C, a 14-h photoperiod, and a photosynthetic photon flux density of 500 μmol m^− 2^ s^− 1^. One-week-old seedlings were chosen to carry out the fluorescent microspheres experiment.

#### Application of suspensions of fluorescent particles

Suspensions of fluorescent microspheres with carboxylate-modified surfaces (FluoSpheres®, Life Technologies Corporation, OR, USA) were prepared to be directly used in the treatments. Two particle sizes (0.1 and 1 μm diameter) were chosen to imitate the foliar penetration of dust particles. Specifications of carboxylate-modified fluorospheres are shown in *the* Additional file 2: Appendix Table. A 10-ml droplet of the suspension was applied to the abaxial surface between the leaf veins of a 1-week-old seedling. Two droplets were applied per leaflet. To avoid rapid drying of surface suspensions, the seedlings in their culturing vessels were put into airtight polythene boxes. The boxes were transparent to light and filled with distilled water (2 cm) in the bottom. Thus, the ambient RH in the box was maintained at 100%. Each particle treatment included 5 to 7 leaves.

#### Removal of residual particles from the leaves

After 2–3 weeks, droplets on the leaf surface were blotted with filter paper. To remove the residues, adhesive tape (‘tesafilm’; Tesa, Hamburg, Germany) was repeatedly affixed to and stripped off the leaf surface five times and a layer of plasticine was used on leaf surface and stripped off immediately. The leaf surface was observed with fluorescence microscopy to confirm the removal of particles. The treatment with plasticine was repeated until no visible particle matter remained.

#### Confocal laser scanning microscopy (CLSM)

Leaf samples were examined with a LSM 800 equipped with an Axio Observer microscope (*Zeiss*, Oberkochen, Germany). The specimens were excited at 650 nm or at 548 nm using appropriate filter combinations. When fluorescence signals were strongest, specimen images were collected about 20–40 μm from the leaf surface. Leaf surface structures were simultaneously visualized by bright-field transmission microscopy. When necessary, colors of the images were adjusted to improve contrasts using imaging systems (Zan 2.1) by ZEISS Company.

### Statistical analysis

One-way analysis of variance (*ANOVA*) was performed to compare the mean differences between the dust accumulation treatments and the control. Post Hoc Tests used Student-Newman-Keuls test (*S-N-K test*) at 5% level. Independent-samples T-test was used to test the difference between two values (before and after high light treatments) also at 5% level. Statistical analysis was performed using *SPSS* (13.0).

## Supplementary Information

**Additional file 1: Appendix Figure. **Schematic diagram of dust application.

**Additional file 2: Appendix Table.** Characteristics of fluospheres carboxylate-modified

## Data Availability

All data generated or analyzed during this study are included in this published article and its supplementary information files. The datasets used and/or analyzed during the current study are available from the corresponding author on reasonable request.

## Abbreviations

LD, MD, HD: light, medium and heavy dust accumulation
*P*_N_: Net photosynthetic rates
*g*_s_: Stomatal conductance to water vapor
*C*_i_: Internal CO_2_ concentration
F_v_: Maximal variable fluorescence
φ_Po_: Maximum quantum yield of primary photochemistry
φ_Eo_: Quantum yield of electron transport from Q_A_ to Q_B_
φ_Do_: Quantum yields of energy dissipation
δ_Ro_: Efficiency/probability with which an electron from Q_B_ is transferred until PSI acceptors
*P*_Nmax_: Light-saturated net photosynthetic rate
LCP: Light compensation point
LSP: Light saturation point
SLA: Specific leaf area
CLSM: Confocal laser scanning microscopy

## References

[1] Shao Y (2008). Physics and Modelling of wind erosion.

[2] Prusty BAK, Mishra PC, Azeez PA (2005). Dust accumulation and leaf pigment concentration in vegetation near the national highway at Sambalpur, Orissa, India. Ecotox Environ Safe.

[3] Sæbø A, Popek R, Nawrot B, Hanslin HM, Gawronska H, Gawronski SW (2012). Plant species differences in particulate matter accumulation on leaf surfaces. Sci Total Environ.

[4] Rai A, Kulshreshtha K, Srivastava PK, Mohanty CS (2010). Leaf surface structure alterations due to particulate pollution in some common plants. Environmentalist..

[5] Pereira EG, Oliva MA, Kuki KN, Cambraia J (2009). Photosynthetic changes and oxidative stress caused by iron ore dust deposition in the tropical CAM tree *Clusia hilariana*. Trees-Struct Funct.

[6] Sharifi MR, Gibson AC, Rundel PW (1997). Surface dust impacts on gas exchange in Mojave Desert shrubs. J Appl Ecol.

[7] Naidoo G, Chirkoot D (2004). The effects of coal dust on photosynthetic performance of the mangrove, *Avicennia marina* in Richards Bay, South Africa. Environ Pollut.

[8] Neves NR, Oliva MA, Centeno DC, Costa AC, Ribas RF, Pereira EG (2009). Photosynthesis and oxidative stress in the resting plant species *Eugenia uniflora* L. exposed to simulated acid rain and iron ore dust deposition: potential use in environmental risk assessment. S Total Environ.

[9] Nanos GD, Ilias IF (2007). Effects of inert dust on olive (*Olea europeaea* L.) leaf physiological parameters. Environ Sci Pollut R.

[10] Farmer AM (1993). The effects of dust on vegetation-a review. Environ Pollut.

[11] Vanderstock AM, Latty T, Leonard RJ, Hochuli DF (2019). Mines over matter: effects of foliar particulate matter on the herbivorous insect, *Helicoverpa armigera*. J Appl Entomol.

[12] Arrivabene HP, Souza IC, Co WLO, Conti MM, Wunderlin DA, Milanez CRD (2015). Effect of pollution by particulate iron on the morphoanatomy, histochemistry, and bioaccumulation of three mangrove plant species in Brazil. Chemosphere..

[13] Rai PK (2016). Impacts of particulate matter pollution on plants: implications for environmental biomonitoring. Ecotoxicol Environ Safe.

[14] Karmakar D, Padhy PK (2019). Metals uptake from particulate matter through foliar transfer and their impact on antioxidant enzymes activity of *S. robusta* in a tropical forest, West Bengal, India. Arch Environ Con Tox.

[15] Valladares F, Niinemets Ü (2008). Shade tolerance, a key plant feature of complex nature and consequences. Annu Rev Ecol Evol S.

[16] Gommers CMM, Visser EJW, Onge KRS, Voesenek LACJ, Pierik R (2013). Shade tolerance: when growing tall is not an option. Trends Plant Sci.

[17] Khan SR, Rose RR, Haase DL, Sabin TE (2000). Effects of shade on morphology, chlorophyll concentration, and chlorophyll fluorescence of four Pacific northwest conifer species. New Forest.

[18] Lichtenthaler HK, Babani F, Navrátil M, Buschmann C (2013). Chlorophyll fluorescence kinetics, photosynthetic activity, and pigment composition of blue-shade and half-shade leaves as compared to sun and shade leaves of different trees. Photosynth Res.

[19] Huang D, Wu L, Chen JR, Dong L (2011). Morphological plasticity, photosynthesis and chlorophyll fluorescence of *Athyrium pachyphlebium* at different shade levels. Photosynthetica..

[20] Hirano T, Kiyota M, Aiga I (1995). Physical effects of dust on leaf physiology of cucumber and kidney bean plants. Environ Pollut.

[21] González JA, Prado FE, Piacentini RD (2014). Atmospheric dust accumulation on native and non-native species: effects on gas exchange parameters. J Environ Qual.

[22] Li L, Mu G (2018). Short-term effects of surface dust: alleviating photoinhibition of cotton under high irradiance in the Tarim Basin. Photosynthetica..

[23] Yu WQ, Wang YJ, Wang YQ, Li B, Liu YJ, Liu X (2018). Application of a coupled model of photosynthesis and stomatal conductance for estimating plant physiological response to pollution by fine particulate matter (PM2.5). Environ Sci Pollut R.

[24] Corredor E, Testillano PS, Coronado M, González-Melendi P, Fernández-Pacheco R, Marquina C, Ibarra MR, la Fuente JM, Rubiales D, Pérez-de-Luque A, Risueño MC (2009). Nanoparticle penetration and transport in living pumpkin plants: in situ subcellular identification. BMC Plant Biol.

[25] Strasser RJ, Srivastava A, Tsimilli-Michael M (2004). Analysis of the chlorophyll *a* fluorescence transient. In: Papageorgiou GC, Govindjee, editors. Chlorophyll *a* Fluorescence: A Signature of Photosynthesis, Advances in Photosynthesis and Respiration Series.

[26] Zivcak M, Brestic M, Kalaji HM, Govindjee (2014). Photosynthetic responses of sun- and shade-grown barley leaves to high light: is the lower PSII connectivity in shade leaves associated with protection against excess of light?. Photosynth Res.

[27] Li L, Zhou Z, Liang J, Lv R (2015). *In vivo* evaluation of the high-irradiance effects on PSII activity in photosynthetic stems of *Hexinia polydichotoma*. Photosynthetica..

[28] Li H, Jiang D, Wollenweber B, Dai T, Cao W (2010). Effects of shading on morphology, physiology and grain yield of winter wheat. Eur J Agron.

[29] Mu H, Jiang D, Wollenweber B, Dai T, Jing Q, Cao W (2010). Long-term low radiation decreases leaf photosynthesis, photochemical efficiency and grain yield in winter wheat. J Agron Crop Sci.

[30] Mendes MM, Gazarini LC, Rodrigues ML (2001). Acclimation of *Myrtus communis* to contrasting Mediterranean light environments – effects on structure and chemical composition of foliage and plant water relations. Environ Exp Bot.

[31] Lavinsky AO, Gomes EP, Mielke MS, França S (2014). Photosynthetic acclimation in shade-developed leaves of *Euterpe edulis* Mart (Arecaceae) after long-term expose to high light. Photosynthetica..

[32] Farquhar GD, Sharkey TD (1982). Stomatal conductance and photosynthesis. Ann Rev Plant Physiol.

[33] Kitao M, Hida T, Eguchi N, Tobita H (2016). Light compensation points in shade-grown seedlings of deciduous broadleaf tree species with different successional traits raised under elevated CO_2_. Plant Biol.

[34] Zhao D, Hao Z, Tao J (2012). Effects of shade on plant growth and flower quality in the herbaceous peony (*Paeonia lactiflora* pall.). Plant Physiol Bioch.

[35] De Costa GS, Dalmolin ÂC, Schilling AC, Sanches MC, dos Santos MS, Mielke MS (2019). Physiological and growth strategies of two Cariniana species in response to contrasting light availability. Flora..

[36] Feng Y (2008). Photosynthesis, nitrogen allocation and specific leaf area in invasive *Eupatorium adenophorum* and native *Eupatorium japonicum* grown at different irradiances. Physiol Plantarum..

[37] Cavatte PC, Oliveira ÁAG, Morais LE, Martins SCV, Sanglard LMVP, Damatta FM (2011). Could shading reduce the negative impacts of drought on coffee? A morphophysiological analysis. Physiol Plantarum.

[38] Baldi P, Muthuchelian K, la Porta N (2012). Leaf plasticity to light intensity in Italian cypress (*Cupressus sempervirens* L.): Adaptability of a Mediterranean conifer cultivated in the Alps. J Photoch Photobio B.

[39] Evans JR, Poorter H (2001). Photosynthetic acclimation of plants to growth irradiance: the relative importance of specific leaf area and nitrogen partitioning in maximizing carbon gain. Plant Cell Environ.

[40] Ashton PMS, Berlyn GP (1994). A comparison of leaf physiology and anatomy of *Quercus* (section *Erythrobalanus*-Fagaceae) species in different light environments. Am J Bot.

[41] Ferreira MJ, de Carvalho Gonçalves JF, Ferraz JBS, dos Santos Junior UM, Rennenberg H (2016). Clonal variation in photosynthesis, foliar nutrient concentrations, and photosynthetic nutrient use efficiency in a Brazil nut (*Bertholletia excelsa*) plantation. For Sci.

[42] Gratani L, Covone F, Larcher W (2006). Leaf plasticity in response to light of three evergreen species of the Mediterranean maquis. Trees-Struct Funct..

[43] Poorter H, Nagel OW (2000). The role of biomass allocation in the growth response of plants to different levels of light, CO2, nutrients and water: a quantitative review. Aust J Plant Physiol.

[44] Koike T, Kitao M, Maruyama Y, Mori S, Lei TT (2001). Leaf morphology and photosynthetic adjustments among deciduous broad-leaved trees within the vertical canopy profile. Tree Physiol.

[45] Murchie EH, Horton P (1998). Contrasting patterns of photosynthetic acclimation to the light environment are dependent on the differential expression of the responses to altered irradiance and spectral quality. Plant Cell Environ.

[46] Jiang Y, Carrow RN, Duncan RR (2005). Physiological acclimation of seashore paspalum and bermudagrass to low light. Sci Hortic-Amsterdam.

[47] Buchholz A, Baur P, Schonherr J (1998). Differences among plant species in cuticular permeabilities and solute mobilities are not caused by differential size selectivities. Planta..

[48] Eichert T, Kurtz A, Steiner U, Goldbach HE (2008). Size exclusion limits and lateral heterogeneity of the stomatal foliar uptake pathway for aqueous solutes and water-suspended nanoparticles. Physiol Plantarum..

[49] Burhardt J, Hunsche M. “Breath figures” on leaf surfaces-formation and effects of microscopic leaf wetness. Front Plant Sci. 2013. 10.3389/fpls.2013.00422. 10.3389/fpls.2013.00422 PMC3807045 24167510

[50] Stirbet A, Govindjee (2011). On the relation between the Kautsky effect (chlorophyll *a* fluorescence induction) and photosystem II: basics and applications of the OJIP fluorescence transient. J Photoch Photobio B.

[51] Prado CHBA, de Moraes JAPV (1997). Photosynthetic capacity and specific leaf mass in twenty woody species of Cerrado vegetation under field conditions. Photosynthetica..

[52] Hieke S, Menzel CM, Lüdders P (2002). Effects of light availability on leaf gas exchange and expansion in lychee (*Litchi chinensis*). Tree Physiol.

[53] Marr IL, Suryana N, Lukulay P (1995). Determination of chlorophyll *a* and *b* by simultaneous multi-component spectrophotometry. Fresenius’ J Anal Chem.

