# Supplementary figures and images for: Similar effects as shade tolerance induced by dust accumulation and size penetration of particulates on cotton leaves
Source: BMC Plant Biol. 2021 Mar 23;21:149. doi: 10.1186/s12870-021-02926-6 (PMC7986255; doi:10.1186/s12870-021-02926-6)

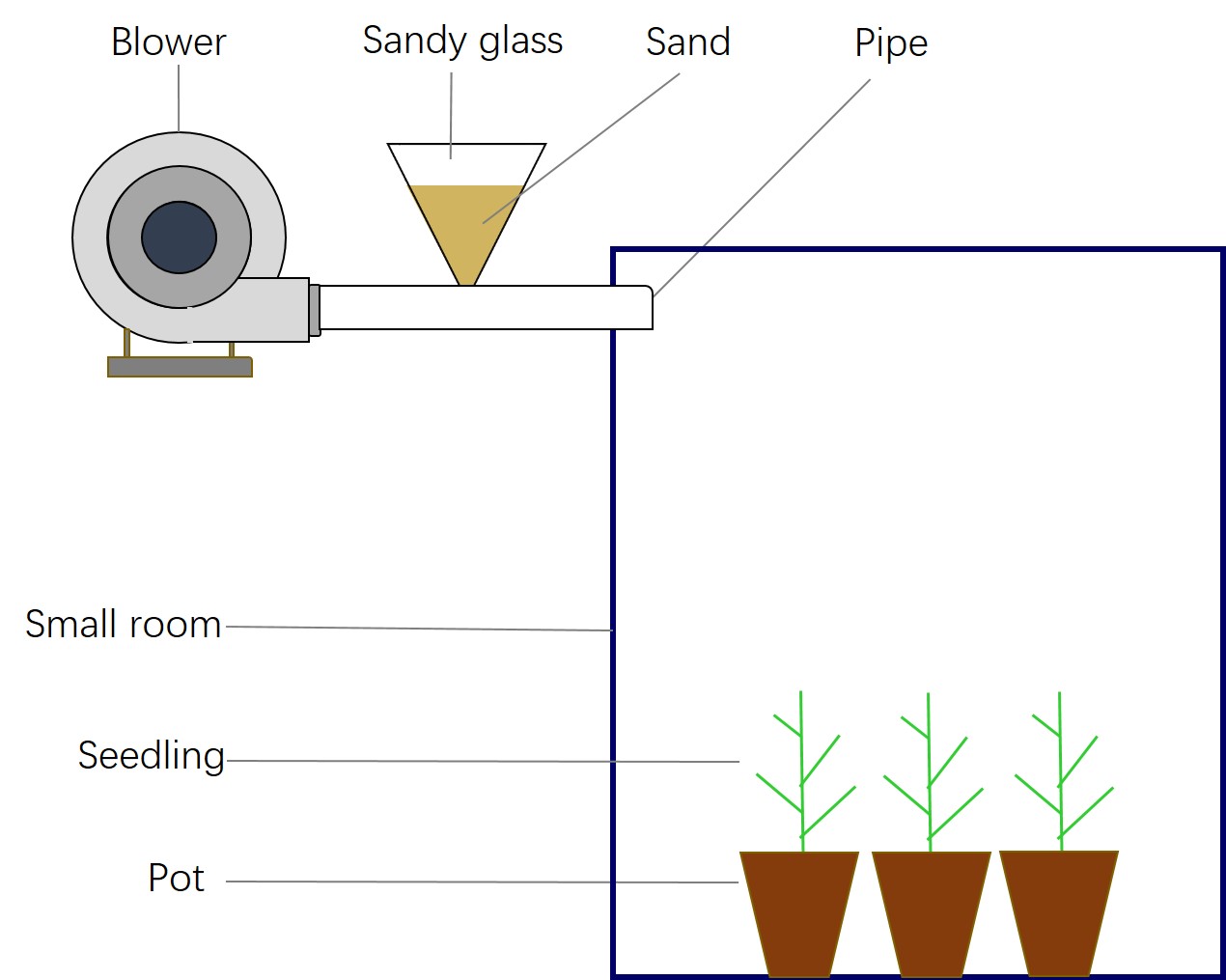

Supplement: Supplementary file 1 — Additional file 1: Appendix Figure. Schematic diagram of dust application. [file 12870_2021_2926_MOESM1_ESM.jpg]
