# Supplementary material for: Similar effects as shade tolerance induced by dust accumulation and size penetration of particulates on cotton leaves
Source: BMC Plant Biol. 2021 Mar 23;21:149. doi: 10.1186/s12870-021-02926-6 (PMC7986255; doi:10.1186/s12870-021-02926-6)
Supplement: Supplementary file 2 — Additional file 2: Appendix Table. Characteristics of fluospheres carboxylate-modified [file 12870_2021_2926_MOESM2_ESM.docx]

*Appendix* Table. Characteristics of fluospheres carboxylate-modified.

| Parameters | F8801 | F8820 |
| --- | --- | --- |
| Diameter (μm)  Surface functional group  Fluorescence  λex/λem (nm)  Concentration (solids)  Liquid phase | 0.099  carboxylic  Red  580-605  2%  Distilled water 2mM azide | 1.0  carboxylic  orange  540-560  2%  2mM Sodium azide |
